# Sculpting Solutions: A Case Report of Resection and Reconstruction in an Aggressive Mandibular Juvenile Ossifying Fibroma and Review of Literature

**DOI:** 10.7759/cureus.79026

**Published:** 2025-02-14

**Authors:** Sujeeth K Shetty, Rishabh Kasrija, Adarsh Choudhary, Amey Hariani, Revati M Kale

**Affiliations:** 1 Department of Oral and Maxillofacial Surgery, Jagadguru Sri Shivarathreeshwara (JSS) Dental College and Hospital, Mysuru, IND

**Keywords:** costochondral graft, fibro-osseous lesions, juvenile, psammomatoid, surgical excision

## Abstract

Juvenile ossifying fibroma (JOF) is a rare, benign, yet locally aggressive fibro-osseous lesion that primarily affects children and adolescents. This case report describes a 10-year-old female patient presenting with a three-month history of progressive swelling on the right lower third of her face, accompanied by trismus. Clinical examination revealed a firm, non-tender swelling extending from the right corner of the mouth to the angle of the mandible, with buccal cortical expansion and vestibular tenderness near the mandibular right second molar. Radiographic imaging demonstrated ill-defined mixed radiolucent lesions extending to the condylar region of the right mandible, prompting a provisional diagnosis of a fibro-osseous lesion, specifically ossifying fibroma. The histopathological evaluation confirmed the psammomatoid variant of JOF, a rare and aggressive subtype that typically occurs in the sinonasal region, making its mandibular presentation unusual. Given the aggressive nature of this variant, surgical resection was performed to minimize recurrence risk. The defect was reconstructed using a costochondral graft, a preferred method due to its adaptability, biocompatibility, and growth potential, particularly in pediatric patients. Costochondral grafts have been historically favored in maxillofacial reconstruction for their ability to mimic native bone structure and support functional restoration. Costochondral grafts have success in treating mandibular defects, especially in growing individuals, as they allow for continued growth and integration with surrounding tissues. This case highlights the importance of early diagnosis, aggressive surgical management, and effective reconstruction in managing rare and aggressive variants of JOF, ensuring both functional and aesthetic outcomes.

## Introduction

Juvenile ossifying fibroma (JOF) is a rare and distinctive benign fibro-osseous lesion that occurs predominantly in the craniofacial bones, especially in children and young adults. It was first described in the literature as a variant of ossifying fibroma, distinguished by its early onset, rapid growth, and locally aggressive behavior [1]. Psammomatoid JOF (PsJOF) was first described by Benjamins in 1938 as "osteoid fibroma with atypical ossification of the frontal sinus." Gögl renamed it "psammomatoid ossifying fibroma" in 1949, and Johnson et al. referred to it as "juvenile active ossifying fibroma" in 1952 [2,3]. JOF has been categorized into two histopathological subtypes, the trabecular type and the psammomatoid type, with the latter being more common in the paranasal sinuses and the former in the jaws [4]. The incidence of JOF is relatively low, accounting for less than 2% of all fibro-osseous lesions, with no significant gender predilection [5]. However, it has a slightly higher prevalence in individuals under 15 years of age [1].

Clinically, JOF presents as a painless, progressively enlarging mass that may cause facial asymmetry, displacement of adjacent teeth, and sometimes paresthesia or pressure effects on surrounding structures [4]. In the mandible, where its occurrence is rare but significant, JOF can lead to functional impairment, such as difficulty in mastication and speech, owing to its aggressive nature and potential to invade surrounding tissues [6]. Radiographically, JOF often appears as a well-demarcated radiolucent to mixed radiolucent-radiopaque lesion with varying degrees of calcification and cortical expansion [7].

Histopathologically, JOF is characterized by a cellular fibroblastic stroma containing irregular trabeculae of immature woven bone (trabecular type) or psammoma-like calcifications (psammomatoid type) [8]. Its aggressive behavior and histological features differentiate it from other fibro-osseous lesions, such as fibrous dysplasia and conventional ossifying fibroma [9]. Differential diagnoses include ameloblastoma, odontogenic keratocyst, osteoblastoma, and aneurysmal bone cyst [1,4].

The importance of managing JOF in the mandible lies in its potential for local recurrence and significant morbidity if inadequately treated. Surgical excision remains the treatment of choice, ranging from conservative enucleation and curettage to more aggressive approaches, such as segmental resection, depending on the extent of the lesion [10]. However, JOF is associated with a high recurrence rate, ranging from 30% to 58%, particularly in cases of incomplete excision. Complications may include facial deformity, functional impairment, and difficulty in reconstructive procedures after extensive resection [11]. Early diagnosis and appropriate treatment are critical to prevent recurrence and preserve function and aesthetics. Postoperative monitoring is essential due to the high recurrence rate associated with incomplete excision. This case is unique as it represents a rare instance of the psammomatoid variant of JOF occurring in the mandible of a 10-year-old, an uncommon presentation in both this age group and anatomical location.

## Case presentation

A 10-year-old female patient presented to the Department of Oral and Maxillofacial Surgery at Jagadguru Sri Shivarathreeshwara (JSS) Dental College and Hospital, Mysuru, with the chief complaint of swelling on the right lower third of her face, persisting for three months. The swelling was of acute onset and gradually progressive, accompanied by trismus. The patient reported mild symptom relief with over-the-counter analgesics.

**Figure 1:**
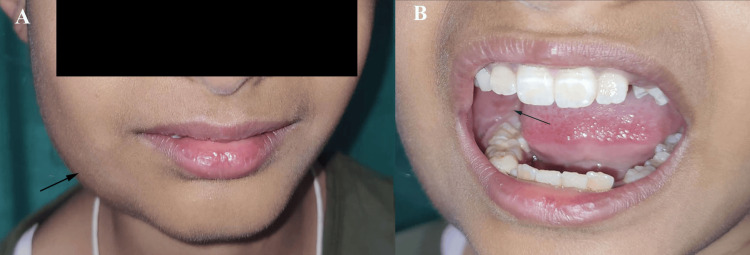
Clinical presentation of mandibular swelling on the right side: (A) extraoral and (B) intraoral All images are original and obtained by the authors

On general examination, the patient appeared well-nourished and in no acute distress. Vital signs were within normal limits: temperature 98.6°F, pulse rate 84 beats per minute, respiratory rate 18 breaths per minute, and blood pressure 108/70 mmHg. No systemic abnormalities were noted. On clinical examination of the face, a firm, non-fluctuant, and non-tender diffuse swelling was observed on the right lower third of the face (Figure 1A).

**Figure 2:**
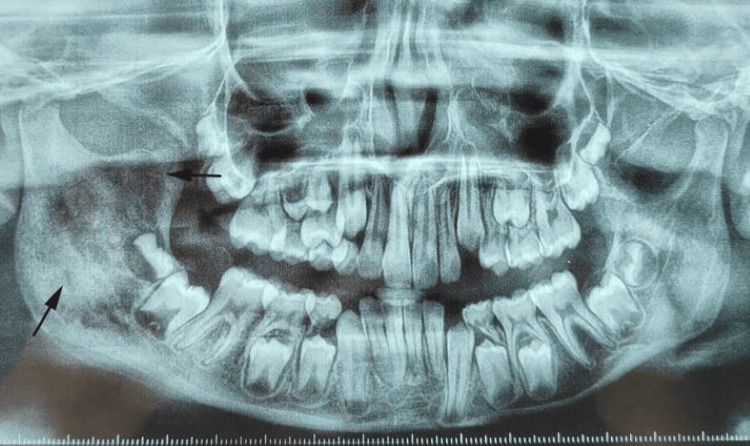
OPG showing mixed radiolucency of the ramus on the right side of the mandible OPG: orthopantomogram The current image is original and obtained by the authors

The swelling extended anteroposteriorly from the right corner of the mouth to the right angle of the mandible and superoinferiorly from the ala-tragus line to the inferior border of the mandible. Intraorally, there was buccal cortical plate expansion in the region of the mandibular right first molar (#46), with associated vestibular tenderness (Figure 1B). The overlying mucosa appeared intact, and there was no evidence of ulceration or discharge. Neurological examination revealed no sensory or motor deficits. Orthopantomogram (OPG) revealed ill-defined mixed radiolucent lesions involving the ramus on the right side of the mandible, extending to the condylar region (Figure 2).

**Figure 3:**
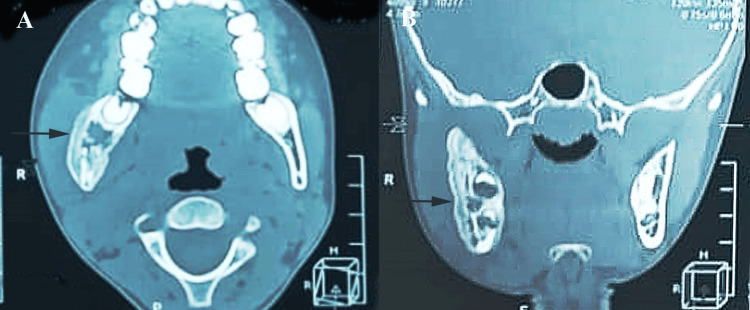
CECT scans revealing multiple ill-defined radiolucent lesions: (A) axial view and (B) oblique view CECT: contrast-enhanced computed tomography

The contrast-enhanced computed tomography (CECT) scans revealed multiple ill-defined radiolucent lesions involving the right hemimandible, extending to the condylar region (Figure 3A, 3B).

The differential diagnosis comprised ameloblastoma, Pindborg tumor, and ossifying fibroma. Since the patient was carrying an incisional biopsy report done by a private practitioner with the diagnosis of ossifying fibroma, the treatment was planned on those lines.

**Figure 4:**
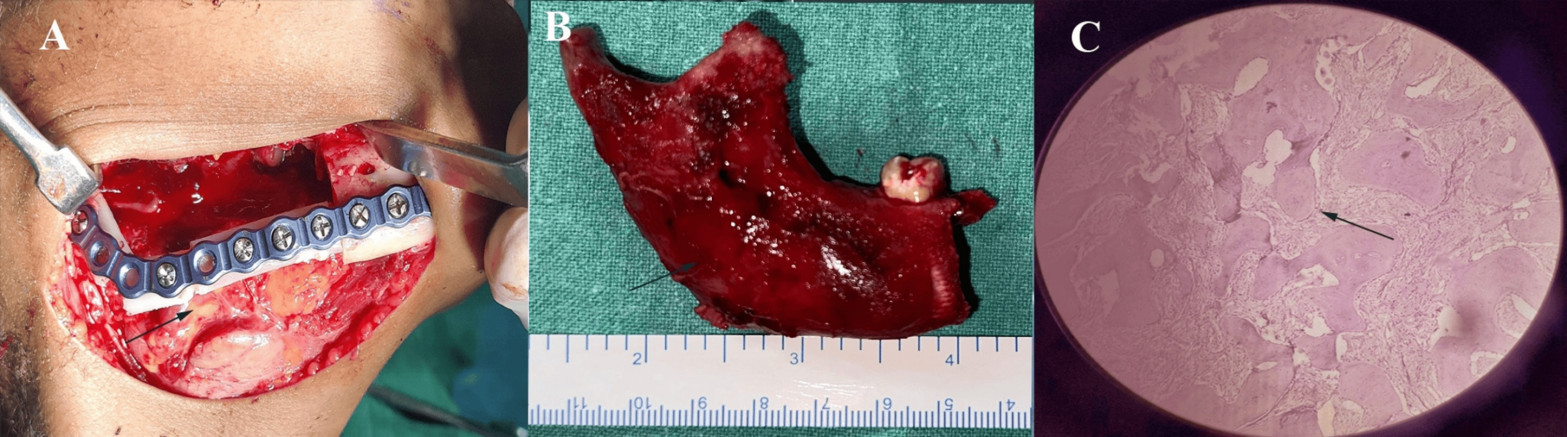
(A) Surgical excision of the lesion and placement of the costochondral graft. (B) Excised lesion. (C) Histopathology showing psammomatoid variety bony trabeculae in fibrocellular background Histopathology: hematoxylin and eosin-stained section at 40× magnification All images are original and obtained by the authors

With the aggressive nature of the lesion, a right hemimandibulectomy with primary reconstruction using a costochondral graft under general anesthesia was planned. Access was achieved via a combination of submandibular, mandibular vestibular, and ascending ramal incisions (Figure 4A).

The tumor, along with the involved portion of the mandible, including the condyle, was completely resected with safe margins (Figure 4B).

Care was taken to preserve the inferior alveolar nerve and overlying periosteum. A 7 cm mandibular defect was reconstructed using a costochondral graft harvested from the seventh rib. A negative Valsalva maneuver confirmed the absence of pleural perforation. The graft was meticulously shaped and secured with a 2.7 mm reconstruction plate, ensuring alignment and stability. Post-reconstruction, occlusion was verified, mandibular movements were evaluated, and the surgical site was closed in layers. Histopathological evaluation (Figure 4C) of the resected specimen confirmed JOF (psammomatoid variant).

**Figure 5:**
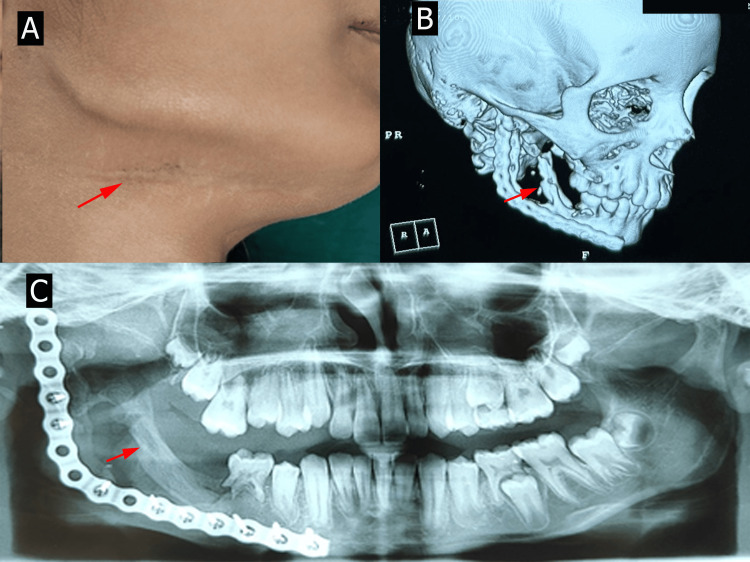
Postoperative follow-up showing (A) healed sutures and (B) satisfactory graft integration and cortical bone remodeling after six months on CBCT and (C) after one year on OPG CBCT: cone-beam computed tomography; OPG: orthopantomogram All images are original and obtained by the authors

The patient has been on regular follow-ups (Figure 5A), with CT imaging after six months (Figure 5B) and OPG after one year (Figure 5C) postoperatively demonstrating satisfactory graft integration and cortical bone remodeling, particularly in the condylar region. The patient exhibited good functional and aesthetic outcomes, with no evidence of recurrence or complications till date.

## Discussion

JOF was categorized into two subtypes, JOF-WHO type and JOF-PO (psammoma-like ossicles) type, based on differences in the age of presentation. The JOF-WHO type has a mean occurrence age of 11.8 years, while the JOF-PO type typically presents at 22.6 years [2]. Similarly, El-Mofty classified JOF into trabecular JOF (TrJOF) and PsJOF based on histological features and age-specific trends [3]. TrJOF usually occurs between 8.5 and 12 years of age, whereas PsJOF is more common in individuals aged 16-33 years. Furthermore, inadequate surgical management of JOF is linked to a high recurrence rate, with recurrences often occurring early and being more aggressive than the initial lesions [6].

Clinically, the patient's primary complaint of progressive swelling accompanied by trismus aligns with the known aggressive behavior of JOF, particularly in the mandibular region [5,6]. Approximately 75% of PsJOFs occur in the orbit, paranasal sinuses, and calvaria, while only around 25% of cases are found in the maxilla or mandible [12]. The absence of systemic symptoms and the localized nature of the swelling are consistent with previous reports in the literature, which emphasize that JOF typically presents as an asymptomatic mass, often discovered due to swelling or associated facial deformity [1,10]. The involvement of the mandibular body and condyle, as seen in this case, is noteworthy because mandibular lesions often result in cortical expansion and facial asymmetry, as observed in this patient. Ong and Siar described JOF as a progressively growing lesion that, if untreated, can cause significant facial deformity due to its enlargement. They documented a case of a 15-year-old male patient with a large cemento-ossifying fibroma in the left mandible, where delayed treatment led to a spontaneous mandibular fracture [13].

Radiographically, the ill-defined radiolucent lesion extending to the condylar region is a characteristic of JOF. PsJOF, in particular, often appears as a mixed radiolucent-radiopaque lesion due to its propensity for calcified matrix deposition. The observed findings align with studies that highlight the aggressive radiographic features of PsJOFs, including cortical thinning and expansion [12]. Differential diagnoses such as fibrous dysplasia, osteosarcoma, or other fibro-osseous lesions must be considered; however, the biopsy and histopathological confirmation of psammomatoid ossicles within a fibrous stroma solidify the diagnosis [7,9]. Fibrous dysplasia exhibits a "ground-glass" radiographic appearance, lacks well-demarcated borders, and blends with the surrounding bone, unlike JOF's circumscribed radiolucent or mixed appearance [9]. Osteosarcoma, a malignant tumor, presents aggressive periosteal reactions, such as a "sunburst" pattern, rapid growth, and associated pain, features absent in benign JOF [7]. Accurate differentiation relies on correlating clinical, radiographic, and histopathological findings to ensure an appropriate diagnosis of JOF.

Histopathologically, the presence of psammomatoid bodies is a hallmark of this variant of JOF; however, the mandibular location of the lesion makes this case unusual, as PsJOF predominantly occurs in the maxilla [1,2]. These structures, along with the cellular fibrous stroma, differentiate JOF from other fibro-osseous lesions such as cemento-ossifying fibroma, which typically lacks this distinct histologic feature [3].

Enucleation or curettage has been recommended as a therapeutic modality for this lesion in various studies. However, this approach was deemed unsuitable in this case due to the extensive size of the lesion, its aggressive growth behavior, and the significant recurrence rates reported in the literature (38-58%) associated with the conservative management of similar pathologies [14]. The aggressive surgical approach in this case, involving hemimandibulectomy and immediate costochondral graft reconstruction, reflects the need for complete resection to minimize recurrence. Fauvel et al. reported a case of a five-year-old boy with a large mandibular TrJOF who underwent mandibulectomy with periosteal preservation and immediate reconstruction using a costal graft via an intraoral approach. The authors observed significant spontaneous mandibular regeneration within a year, with mandibular height increasing from 41.5% to 75.2% and width from 34.4% to 82.8% compared to the unaffected side. They concluded that the costal graft provided structural support, stabilized bone fragments, and prevented soft tissue prolapse, highlighting the importance of periosteal conservation in pediatric reconstruction [15]. The literature supports such radical surgical management for JOF, given its high recurrence rates if incompletely excised [11]. The use of a costochondral graft ensured the restoration of mandibular continuity and condylar function, and its biological compatibility, growth potential, and ability to mimic the natural architecture of the mandible make it ideal for pediatric patients [16,17]. Additionally, costochondral grafts integrate well with surrounding tissues, promoting long-term functional and aesthetic outcomes. However, complications can arise, including graft resorption, overgrowth, infection, or donor site morbidity [17,18]. Improper alignment or fixation may also lead to occlusal issues or limited mandibular movement [16]. Careful surgical planning, precise technique, and regular follow-up are essential to mitigate these potential complications. In the present case, follow-up imaging confirms satisfactory graft integration and remodeling.

## Conclusions

The present case report highlights the rare occurrence of PsJOF in the mandible at a young age. Apt and early diagnosis supplemented by effective treatment requires a thorough evaluation of clinical presentation, imaging characteristics, and histopathological findings in JOF. It is treated primarily through surgical removal, with inadequate resection increasing the risk of recurrence. Given its locally aggressive behavior and significant recurrence potential, ongoing long-term monitoring is essential. In pediatric patients, costochondral grafts provide an effective reconstructive solution due to their adaptability and growth potential.
